# (*E*)-Methyl *N*′-(2-furylmethyl­ene)­hydrazinecarboxyl­ate

**DOI:** 10.1107/S1600536808033825

**Published:** 2008-10-22

**Authors:** Lu-Ping Lv, Yong-Zhao Zhang, Xiao-Min Ding, Wen-Bo Yu, Xian-Chao Hu

**Affiliations:** a Department of Chemical Engineering, Hangzhou Vocational and Technical College, Hangzhou 310018, People’s Republic of China; b Zhejiang Xinan Chemical Industrial Group Co. Ltd., Jiande 311604, People’s Republic of China; c Research Center of Analysis and Measurement, Zhejiang University of Technology, Hangzhou 310014, People’s Republic of China

## Abstract

The title compound, C_7_H_8_N_2_O_3_, crystallizes with two independent but essentially identical mol­ecules in the asymmetric unit. Each mol­ecule adopts a *trans* configuration with respect to the C=N bond. The hydrazinecarboxyl­ate group is twisted from the furan ring by 7.78 (13)° in one mol­ecule and by 7.01 (17)° in the other. In the crystal structure, mol­ecules are linked into chains running along [010] by bifurcated N—H⋯(N,O) and N—H⋯O hydrogen bonds. In addition, weak C—H⋯O inter­actions and an O⋯C short contact [2.896 (3) Å] are observed.

## Related literature

For general background, see: Parashar *et al.* (1988 ▶); Hadjoudis *et al.* (1987 ▶); Borg *et al.* (1999 ▶); Kahwa *et al.* (1986 ▶); Santos *et al.* (2001 ▶). For a related structure, see: Shang *et al.* (2007 ▶).
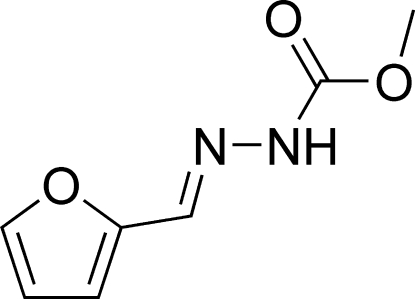


## Experimental

### 

#### Crystal data

C_7_H_8_N_2_O_3_
*M* *_r_* = 168.15
Monoclinic, 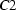

*a* = 14.9185 (17) Å
*b* = 7.8124 (9) Å
*c* = 15.1299 (19) Å
β = 105.251 (7)°
*V* = 1701.3 (4) Å^3^
*Z* = 8
Mo *K*α radiation
μ = 0.10 mm^−1^
*T* = 193 (2) K
0.19 × 0.17 × 0.16 mm

#### Data collection

Bruker SMART CCD area-detector diffractometer
Absorption correction: multi-scan (*SADABS*; Bruker, 2002 ▶) *T*_min_ = 0.978, *T*_max_ = 0.982
4679 measured reflections
1601 independent reflections
1399 reflections with *I* > 2σ(*I*)
*R*_int_ = 0.020

#### Refinement

*R*[*F* ^2^ > 2σ(*F* ^2^)] = 0.031
*wR*(*F* ^2^) = 0.085
*S* = 1.07
1601 reflections
218 parameters
1 restraint
H-atom parameters constrained
Δρ_max_ = 0.10 e Å^−3^
Δρ_min_ = −0.09 e Å^−3^

### 

Data collection: *SMART* (Bruker, 2002 ▶); cell refinement: *SAINT* (Bruker, 2002 ▶); data reduction: *SAINT*; program(s) used to solve structure: *SHELXS97* (Sheldrick, 2008 ▶); program(s) used to refine structure: *SHELXL97* (Sheldrick, 2008 ▶); molecular graphics: *SHELXTL* (Sheldrick, 2008 ▶); software used to prepare material for publication: *SHELXTL*.

## Supplementary Material

Crystal structure: contains datablocks I, global. DOI: 10.1107/S1600536808033825/ci2688sup1.cif

Structure factors: contains datablocks I. DOI: 10.1107/S1600536808033825/ci2688Isup2.hkl

Additional supplementary materials:  crystallographic information; 3D view; checkCIF report

## Figures and Tables

**Table 1:** Hydrogen-bond geometry (Å, °)

*D*—H⋯*A*	*D*—H	H⋯*A*	*D*⋯*A*	*D*—H⋯*A*
N2—H2*A*⋯O5	0.86	2.36	3.138 (3)	151
N2—H2*A*⋯N3	0.86	2.52	3.242 (3)	141
N4—H4⋯O2^i^	0.86	2.11	2.913 (3)	156
C2—H2⋯O5^ii^	0.93	2.60	3.521 (4)	172
C10—H10⋯O5^iii^	0.93	2.59	3.508 (4)	171

Symmetry codes: (i) 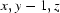; (ii) 
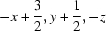
; (iii) 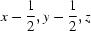.

**Fig. 1.**
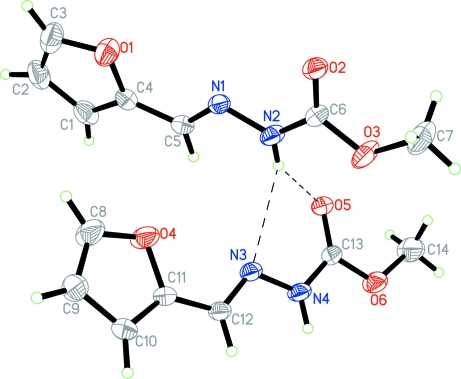
The asymmetric unit of the title compound. Displacement ellipsoids are drawn at the 50% probability level. Dashed lines indicate hydrogen bonds.

**Fig. 2.**
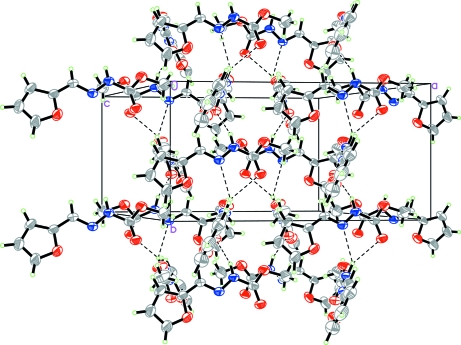
Crystal packing of the title compound. Hydrogen bonds are shown as dashed lines.

## supplementary crystallographic information

### Comment

Benzaldehydehydrazone derivatives have attracted much attention due to their pharmacological activity (Parashar *et al.*, 1988) and their photochromic properties (Hadjoudis *et al.*, 1987). They are important intermidiates of 1,3,4-oxadiazoles, which have been reported to be versatile compounds with many interesting properties (Borg *et al.*, 1999). Metal complexes based on Schiff bases have received considerable attention because they can be utilized as model compounds of active centres in various proteins and enzymes (Kahwa *et al.*, 1986; Santos *et al.*, 2001). We report here the crystal structure of the title compound (Fig. 1).

The title compound contains two independent, but essentially identical molecules in the asymmetric unit. Each independent molecule adopts a *trans* configuration with respect to the C═N bond. The N1/N2/O2/O3/C6/C7 and N3/N4/O5/O6/C13/C14 planes form dihedral angles of 7.78 (13) and 7.01 (17)°, respectively, with the O1/C1–C4 and O4/C8–C11 planes. The dihedral angle between the two independent furan rings is 85.17 (11)°. The bond lengths and angles are comparable to those observed for methyl *N*'-[(*E*)-4-methoxybenzylidene]hydrazinecarboxylate (Shang *et al.*, 2007).

In the crystal structure, the molecules are linked into chains running along the [010] by N—H···O and N—H···N hydrogen bonds (Table 1 and Fig.2). In addition, weak C—H···O interactions and an O4···C5 short contact [2.896 (3) Å] are also observed.

### Experimental

Furfuraldehyde (0.96 g, 0.01 mol) and methyl hydrazinecarboxylate (0.90 g, 0.01 mol) were dissolved in stirred methanol (20 ml) and left for 3 h at room temperature. The resulting solid was filtered off and recrystallized from ethanol to give the title compound in 85% yield. Single crystals suitable for X-ray analysis were obtained by slow evaporation of an ethanol solution at room temperature (m.p. 408–413 K).

### Refinement

H atoms were positioned geometrically (N—H = 0.86 Å and C—H = 0.93 or 0.96 Å) and refined using a riding model, with *U*_iso_(H) = 1.2*U*_eq_(C,N) and 1.5*U*_eq_(C_methyl_). In the absence of significant anomalous scattering effects, Friedel pairs were averaged.

### Crystal data

**Table:**

C_7_H_8_N_2_O_3_	*F*(000) = 704
*M**_r_* = 168.15	*D*_x_ = 1.313 Mg m^−^^3^
Monoclinic, *C*2	Mo *K*α radiation, λ = 0.71073 Å
Hall symbol: C 2y	Cell parameters from 1601 reflections
*a* = 14.9185 (17) Å	θ = 1.4–25.0°
*b* = 7.8124 (9) Å	µ = 0.10 mm^−^^1^
*c* = 15.1299 (19) Å	*T* = 193 K
β = 105.251 (7)°	Block, colourless
*V* = 1701.3 (4) Å^3^	0.19 × 0.17 × 0.16 mm
*Z* = 8	

### Data collection

**Table:**

Bruker SMART CCD area-detector diffractometer	1601 independent reflections
Radiation source: fine-focus sealed tube	1399 reflections with *I* > 2σ(*I*)
graphite	*R*_int_ = 0.020
φ and ω scans	θ_max_ = 25.0°, θ_min_ = 1.4°
Absorption correction: multi-scan (SADABS; Bruker, 2002)	*h* = −17→17
*T*_min_ = 0.978, *T*_max_ = 0.982	*k* = −9→8
4679 measured reflections	*l* = −17→16

### Refinement

**Table:**

Refinement on *F*^2^	Secondary atom site location: difference Fourier map
Least-squares matrix: full	Hydrogen site location: inferred from neighbouring sites
*R*[*F*^2^ > 2σ(*F*^2^)] = 0.031	H-atom parameters constrained
*wR*(*F*^2^) = 0.085	*w* = 1/[σ^2^(*F*_o_^2^) + (0.0464*P*)^2^ + 0.2178*P*] where *P* = (*F*_o_^2^ + 2*F*_c_^2^)/3
*S* = 1.07	(Δ/σ)_max_ = 0.001
1601 reflections	Δρ_max_ = 0.10 e Å^−^^3^
218 parameters	Δρ_min_ = −0.09 e Å^−^^3^
1 restraint	Extinction correction: SHELXL97 (Sheldrick, 2008), Fc^*^=kFc[1+0.001xFc^2^λ^3^/sin(2θ)]^-1/4^
Primary atom site location: structure-invariant direct methods	Extinction coefficient: 0.0051 (9)

### Special details

**Table:**

Geometry. All e.s.d.'s (except the e.s.d. in the dihedral angle between two l.s. planes) are estimated using the full covariance matrix. The cell e.s.d.'s are taken into account individually in the estimation of e.s.d.'s in distances, angles and torsion angles; correlations between e.s.d.'s in cell parameters are only used when they are defined by crystal symmetry. An approximate (isotropic) treatment of cell e.s.d.'s is used for estimating e.s.d.'s involving l.s. planes.
Refinement. Refinement of *F*^2^ against ALL reflections. The weighted *R*-factor *wR* and goodness of fit *S* are based on *F*^2^, conventional *R*-factors *R* are based on *F*, with *F* set to zero for negative *F*^2^. The threshold expression of *F*^2^ > σ(*F*^2^) is used only for calculating *R*-factors(gt) *etc*. and is not relevant to the choice of reflections for refinement. *R*-factors based on *F*^2^ are statistically about twice as large as those based on *F*, and *R*- factors based on ALL data will be even larger.

### Fractional atomic coordinates and isotropic or equivalent isotropic displacement parameters (Å^2^)

**Table:**

	*x*	*y*	*z*	*U*_iso_*/*U*_eq_	
C1	0.6802 (2)	0.5242 (5)	−0.0094 (2)	0.0834 (9)	
H1	0.6985	0.4241	−0.0330	0.100*	
C2	0.6239 (2)	0.6570 (6)	−0.0595 (2)	0.0971 (12)	
H2	0.5969	0.6590	−0.1223	0.117*	
C3	0.6170 (2)	0.7771 (6)	0.0000 (2)	0.1020 (12)	
H3	0.5850	0.8797	−0.0152	0.122*	
C4	0.70165 (17)	0.5730 (4)	0.07936 (17)	0.0617 (7)	
C5	0.75360 (16)	0.4883 (3)	0.16066 (17)	0.0583 (6)	
H5	0.7769	0.3792	0.1561	0.070*	
C6	0.83278 (17)	0.5270 (4)	0.39638 (18)	0.0600 (6)	
C7	0.8972 (3)	0.4683 (6)	0.5526 (2)	0.1191 (15)	
H7A	0.9269	0.3773	0.5922	0.179*	
H7B	0.8411	0.5010	0.5681	0.179*	
H7C	0.9383	0.5648	0.5600	0.179*	
C8	0.5302 (2)	0.2842 (6)	0.1389 (3)	0.1233 (17)	
H8	0.5139	0.3884	0.1093	0.148*	
C9	0.4711 (2)	0.1714 (5)	0.1540 (3)	0.0977 (11)	
H9	0.4067	0.1801	0.1368	0.117*	
C10	0.52379 (19)	0.0344 (5)	0.2012 (2)	0.0786 (8)	
H10	0.5006	−0.0642	0.2216	0.094*	
C11	0.61376 (17)	0.0724 (3)	0.21148 (18)	0.0615 (7)	
C12	0.69799 (17)	−0.0134 (3)	0.25482 (16)	0.0586 (6)	
H12	0.6952	−0.1248	0.2756	0.070*	
C13	0.93793 (17)	0.0282 (4)	0.32608 (17)	0.0609 (6)	
C14	1.0967 (2)	−0.0296 (6)	0.3959 (3)	0.1240 (16)	
H14A	1.1351	−0.1178	0.4307	0.186*	
H14B	1.1047	0.0743	0.4311	0.186*	
H14C	1.1143	−0.0105	0.3400	0.186*	
N1	0.76861 (13)	0.5587 (3)	0.23906 (14)	0.0552 (5)	
N2	0.81642 (15)	0.4606 (3)	0.31122 (14)	0.0652 (6)	
H2A	0.8355	0.3597	0.3025	0.078*	
N3	0.77726 (14)	0.0577 (3)	0.26606 (14)	0.0596 (5)	
N4	0.85235 (15)	−0.0392 (3)	0.31071 (16)	0.0692 (6)	
H4	0.8447	−0.1414	0.3286	0.083*	
O1	0.66343 (14)	0.7290 (3)	0.08659 (13)	0.0860 (7)	
O2	0.81342 (12)	0.6698 (2)	0.41521 (12)	0.0673 (5)	
O3	0.87484 (17)	0.4105 (3)	0.45826 (13)	0.0932 (7)	
O4	0.61942 (13)	0.2275 (3)	0.17282 (18)	0.1027 (8)	
O5	0.95630 (12)	0.1665 (2)	0.30007 (12)	0.0699 (5)	
O6	0.99994 (13)	−0.0822 (3)	0.37459 (16)	0.0895 (7)	

### Atomic displacement parameters (Å^2^)

**Table:**

	*U*^11^	*U*^22^	*U*^33^	*U*^12^	*U*^13^	*U*^23^
C1	0.0933 (19)	0.090 (2)	0.0653 (18)	−0.024 (2)	0.0181 (15)	−0.0164 (18)
C2	0.090 (2)	0.131 (3)	0.0597 (19)	−0.034 (2)	0.0009 (16)	0.004 (2)
C3	0.097 (2)	0.118 (3)	0.080 (2)	0.012 (2)	0.0033 (18)	0.024 (2)
C4	0.0599 (13)	0.0630 (19)	0.0627 (17)	−0.0120 (13)	0.0170 (11)	−0.0070 (14)
C5	0.0606 (12)	0.0503 (16)	0.0669 (16)	−0.0080 (11)	0.0217 (11)	−0.0033 (13)
C6	0.0688 (14)	0.0525 (16)	0.0598 (16)	0.0034 (12)	0.0191 (11)	0.0131 (13)
C7	0.186 (4)	0.101 (3)	0.063 (2)	0.045 (3)	0.021 (2)	0.022 (2)
C8	0.0559 (16)	0.103 (3)	0.200 (4)	0.0057 (19)	0.014 (2)	0.072 (3)
C9	0.0562 (15)	0.102 (3)	0.129 (3)	−0.0063 (18)	0.0139 (16)	0.034 (3)
C10	0.0716 (16)	0.0723 (18)	0.088 (2)	−0.0234 (16)	0.0139 (14)	0.0125 (16)
C11	0.0673 (15)	0.0484 (16)	0.0684 (16)	−0.0082 (12)	0.0168 (12)	0.0072 (13)
C12	0.0711 (15)	0.0428 (14)	0.0618 (15)	−0.0042 (12)	0.0172 (11)	0.0016 (11)
C13	0.0668 (15)	0.0493 (15)	0.0629 (15)	0.0061 (13)	0.0108 (11)	0.0055 (13)
C14	0.0683 (19)	0.096 (3)	0.185 (4)	0.0045 (19)	−0.007 (2)	0.036 (3)
N1	0.0628 (11)	0.0452 (12)	0.0577 (13)	−0.0017 (9)	0.0159 (9)	0.0039 (10)
N2	0.0882 (15)	0.0433 (11)	0.0659 (14)	0.0111 (11)	0.0237 (11)	0.0071 (11)
N3	0.0637 (12)	0.0448 (12)	0.0693 (13)	0.0024 (10)	0.0158 (10)	0.0078 (10)
N4	0.0663 (12)	0.0437 (11)	0.0940 (16)	0.0038 (10)	0.0148 (11)	0.0182 (12)
O1	0.0971 (14)	0.0878 (16)	0.0700 (13)	0.0182 (12)	0.0162 (10)	0.0062 (11)
O2	0.0868 (12)	0.0485 (11)	0.0639 (11)	0.0061 (10)	0.0154 (9)	0.0031 (9)
O3	0.1460 (19)	0.0685 (15)	0.0641 (12)	0.0357 (14)	0.0256 (12)	0.0230 (11)
O4	0.0575 (10)	0.0748 (14)	0.171 (2)	−0.0016 (10)	0.0218 (12)	0.0520 (15)
O5	0.0733 (10)	0.0521 (11)	0.0783 (12)	−0.0049 (10)	0.0092 (9)	0.0131 (10)
O6	0.0698 (11)	0.0645 (14)	0.1240 (17)	0.0081 (10)	0.0075 (11)	0.0283 (13)

### Geometric parameters (Å, °)

**Table:**

C1—C4	1.351 (4)	C8—H8	0.93
C1—C2	1.421 (6)	C9—C10	1.406 (5)
C1—H1	0.93	C9—H9	0.93
C2—C3	1.322 (6)	C10—C11	1.343 (4)
C2—H2	0.93	C10—H10	0.93
C3—O1	1.364 (4)	C11—O4	1.357 (3)
C3—H3	0.93	C11—C12	1.423 (3)
C4—O1	1.362 (4)	C12—N3	1.277 (3)
C4—C5	1.432 (4)	C12—H12	0.93
C5—N1	1.273 (3)	C13—O5	1.206 (3)
C5—H5	0.93	C13—O6	1.334 (3)
C6—O2	1.206 (4)	C13—N4	1.344 (3)
C6—O3	1.337 (3)	C14—O6	1.454 (4)
C6—N2	1.350 (3)	C14—H14A	0.96
C7—O3	1.450 (4)	C14—H14B	0.96
C7—H7A	0.96	C14—H14C	0.96
C7—H7B	0.96	N1—N2	1.370 (3)
C7—H7C	0.96	N2—H2A	0.86
C8—C9	1.309 (5)	N3—N4	1.373 (3)
C8—O4	1.368 (4)	N4—H4	0.86
			
C4—C1—C2	106.0 (3)	C11—C10—C9	107.4 (3)
C4—C1—H1	127.0	C11—C10—H10	126.3
C2—C1—H1	127.0	C9—C10—H10	126.3
C3—C2—C1	107.2 (3)	C10—C11—O4	108.7 (3)
C3—C2—H2	126.4	C10—C11—C12	133.1 (3)
C1—C2—H2	126.4	O4—C11—C12	118.1 (2)
C2—C3—O1	110.3 (4)	N3—C12—C11	122.1 (2)
C2—C3—H3	124.8	N3—C12—H12	119.0
O1—C3—H3	124.8	C11—C12—H12	119.0
C1—C4—O1	109.7 (3)	O5—C13—O6	125.1 (2)
C1—C4—C5	131.0 (3)	O5—C13—N4	125.6 (2)
O1—C4—C5	119.2 (2)	O6—C13—N4	109.4 (2)
N1—C5—C4	121.6 (2)	O6—C14—H14A	109.5
N1—C5—H5	119.2	O6—C14—H14B	109.5
C4—C5—H5	119.2	H14A—C14—H14B	109.5
O2—C6—O3	124.2 (3)	O6—C14—H14C	109.5
O2—C6—N2	125.9 (2)	H14A—C14—H14C	109.5
O3—C6—N2	109.9 (3)	H14B—C14—H14C	109.5
O3—C7—H7A	109.5	C5—N1—N2	115.3 (2)
O3—C7—H7B	109.5	C6—N2—N1	118.1 (2)
H7A—C7—H7B	109.5	C6—N2—H2A	120.9
O3—C7—H7C	109.5	N1—N2—H2A	120.9
H7A—C7—H7C	109.5	C12—N3—N4	115.6 (2)
H7B—C7—H7C	109.5	C13—N4—N3	118.9 (2)
C9—C8—O4	110.5 (3)	C13—N4—H4	120.5
C9—C8—H8	124.8	N3—N4—H4	120.5
O4—C8—H8	124.8	C4—O1—C3	106.7 (3)
C8—C9—C10	106.8 (3)	C6—O3—C7	114.9 (3)
C8—C9—H9	126.6	C11—O4—C8	106.6 (2)
C10—C9—H9	126.6	C13—O6—C14	116.3 (2)
			
C4—C1—C2—C3	1.7 (4)	C5—N1—N2—C6	−178.6 (2)
C1—C2—C3—O1	−1.6 (4)	C11—C12—N3—N4	178.7 (2)
C2—C1—C4—O1	−1.1 (3)	O5—C13—N4—N3	−3.8 (4)
C2—C1—C4—C5	177.6 (3)	O6—C13—N4—N3	176.4 (2)
C1—C4—C5—N1	178.0 (3)	C12—N3—N4—C13	−179.1 (2)
O1—C4—C5—N1	−3.3 (3)	C1—C4—O1—C3	0.2 (3)
O4—C8—C9—C10	0.8 (6)	C5—C4—O1—C3	−178.8 (2)
C8—C9—C10—C11	−0.6 (5)	C2—C3—O1—C4	0.9 (4)
C9—C10—C11—O4	0.1 (4)	O2—C6—O3—C7	−0.4 (4)
C9—C10—C11—C12	178.3 (3)	N2—C6—O3—C7	179.2 (3)
C10—C11—C12—N3	−171.0 (3)	C10—C11—O4—C8	0.3 (4)
O4—C11—C12—N3	7.0 (4)	C12—C11—O4—C8	−178.1 (3)
C4—C5—N1—N2	177.8 (2)	C9—C8—O4—C11	−0.7 (5)
O2—C6—N2—N1	−4.2 (4)	O5—C13—O6—C14	−0.8 (5)
O3—C6—N2—N1	176.2 (2)	N4—C13—O6—C14	179.0 (3)

### Hydrogen-bond geometry (Å, °)

**Table:**

*D*—H···*A*	*D*—H	H···*A*	*D*···*A*	*D*—H···*A*
N2—H2A···O5	0.86	2.36	3.138 (3)	151
N2—H2A···N3	0.86	2.52	3.242 (3)	141
N4—H4···O2^i^	0.86	2.11	2.913 (3)	156
C2—H2···O5^ii^	0.93	2.60	3.521 (4)	172
C10—H10···O5^iii^	0.93	2.59	3.508 (4)	171

Symmetry codes: (i) *x*, *y*−1, *z*; (ii) −*x*+3/2, *y*+1/2, −*z*; (iii) *x*−1/2, *y*−1/2, *z*.

## References

[bb1] Borg, S., Vollinga, R. C., Labarre, M., Payza, K., Terenius, L. & Luthman, K. (1999). *J. Med. Chem.* **42**, 4331–4342. 10.1021/jm990197+ 10543877

[bb2] Bruker (2002). *SADABS*, *SMART* and *SAINT* Bruker AXS Inc., Madison, Wisconsin, USA.

[bb3] Hadjoudis, E., Vittorakis, M. & Moustakali-Mavridis, J. (1987). *Tetrahedron*, **43**, 1345–1360.

[bb4] Kahwa, I. A., Selbin, J., Hsieh, T. Y. & Laine, R. A. (1986). *Inorg. Chim. Acta*, **151**, 201–208.

[bb5] Parashar, R. K., Sharma, R. C., Kumar, A. & Mohanm, G. (1988). *Inorg. Chim. Acta*, **151**, 201–208.

[bb6] Santos, M. L. P., Bagatin, I. A., Pereira, E. M. & Ferreira, A. M. D. C. (2001). *J. Chem. Soc. Dalton Trans.* pp. 838–844.

[bb7] Shang, Z.-H., Zhang, H.-L. & Ding, Y. (2007). *Acta Cryst.* E**63**, o3394.

[bb8] Sheldrick, G. M. (2008). *Acta Cryst.* A**64**, 112–122. 10.1107/S0108767307043930 18156677

